# Tetralogy of Fallot

**DOI:** 10.4103/0974-2069.43880

**Published:** 2008

**Authors:** Bhava R. J. Kannan

**Affiliations:** Consultant Interventional and Pediatric Cardiologist, Vadamalayan Hospitals, Madurai, India

**Keywords:** Balloon valvotomy, tetralogy of Fallot, hemodynamics

## CASE HISTORY

A seven year old girl presented with exertional syncope. She was suspected to have a heart disease at the age of 2 years but did not undergo any further evaluation. On examination, she had quiet precordium, single second sound with 1/6 ejection systolic murmur. Her systemic oxygen saturation was 65% and hemoglobin was 22g%. Echocardiography revealed tetralogy of Fallot (TOF) with severe infundibular and valvar pulmonary stenosis. The branch pulmonary arteries were confluent but the right pulmonary artery (7mm) was hypoplastic as compared to the left pulmonary artery (12.5mm). Total intracardiac repair could not be done due to financial reasons and hence it was decided to palliate her with balloon pulmonary valvotomy.

**Figure 1:**
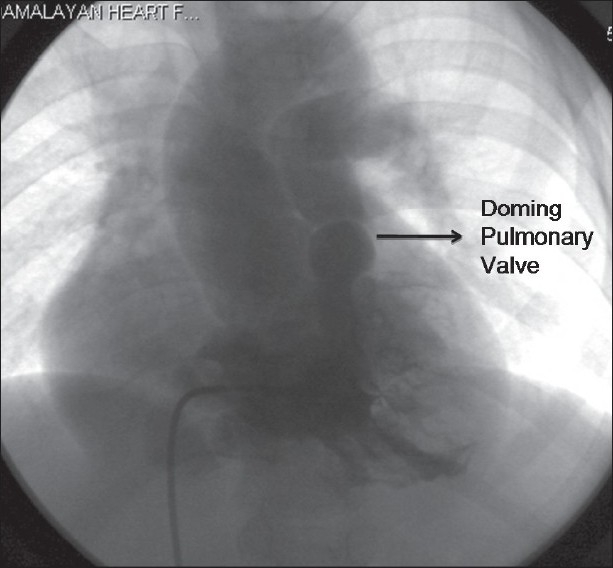
Right ventricular angiogram done in postero anterior view with 15 degree cranial angulation showing infundibular narrowing and doming pulmonary valve

**Figure 2:**
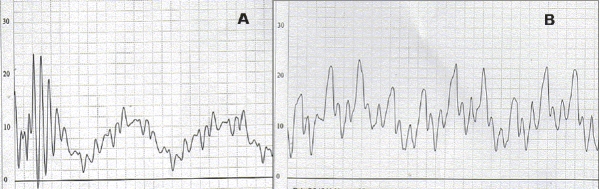
Pulmonary artery pressure tracings. (A) baseline pressure trace showing low pressure with poor waveforms. (B) following balloon pulmonary valvotomy, there is increase in the pressure with more distinct waveforms

Cardiac catheterization was done under local anesthesia and intravenous sedation with midazolam. Special attention was paid to her hydration before bringing her to the catheterization laboratory. The pressure data is summarized in Table 1. Right ventricular outflow tract (RVOT) gradient was 110mmHg. Right ventricular angiogram revealed doming pulmonary valve with a diminished antegrade flow [Figure 1]. The basal pulmonary artery pressure tracing showed poor waveform with a mean pressure of 8mmHg [Figure 2A]. Pulmonary annulus measured 16.6mm. Pulmonary valvotomy was done with a 20mm Tyshak balloon with complete abolition of the waist. Repeat pressure studies showed normal waveform in the pulmonary artery pressure tracing with a systolic pressure of 20mmHg [Figure 2B]. Severe RVOT obstruction persisted with a gradient of 100mHg. However, the systemic saturation increased to 88% with appearance of a 3/6 ejection systolic murmur. Essentially, a cyanotic TOF child was converted to ‘pink TOF’ by balloon pulmonary valvotomy.

**Table 1:** Summary of pressure data before and after balloon pulmonary valvotomy

	Basal pressure (mmHg)	Final pressure (mmHg)
Right atrium	6	8
Right ventricle	116	120
Pulmonary artery	(6)	20/5 (11)
Pressure gradient	110	100

## DISCUSSION

**Figure 3:**
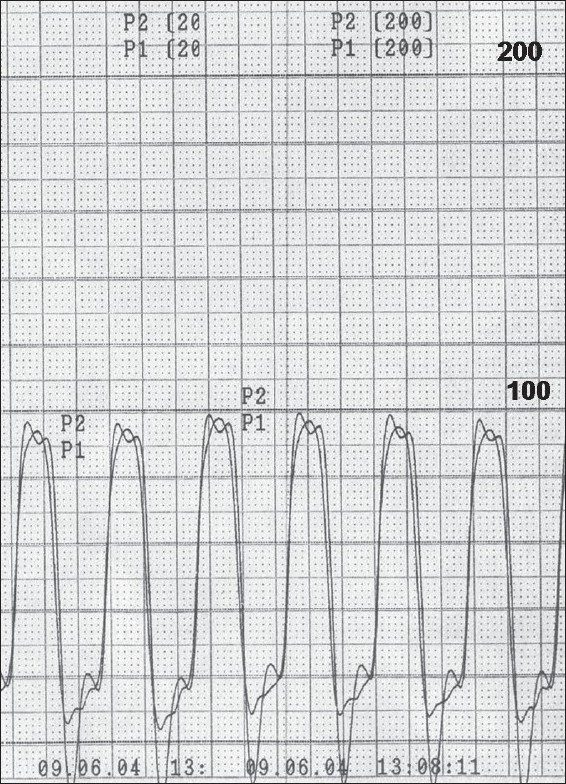
Simultaneous pressure tracings from right and left ventricle in a child with TOF. Both ventricles have same systolic pressure and similar configuration

**Figure 4:**
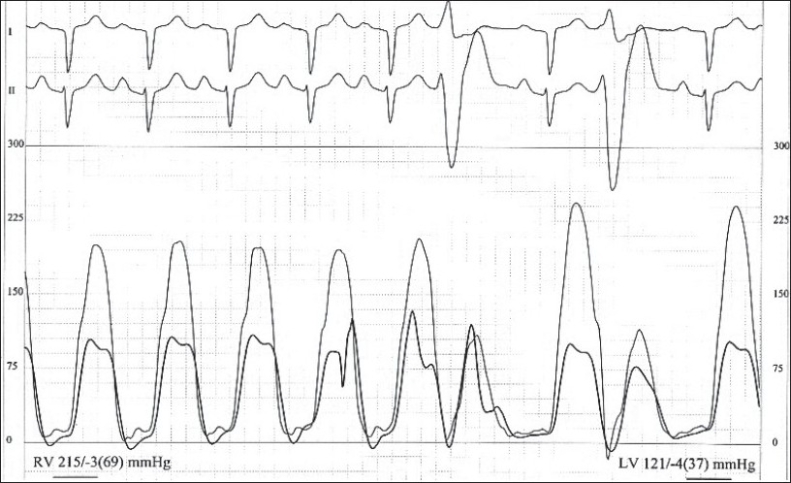
Shows simultaneous right and left ventricular pressure tracings in a child with tetralogy with Fallot and restrictive VSD. Note the triangular pattern of the right ventricular waveform with post extrasystolic potentiation

TOF patients can present with severe cyanosis or can be asymptomatic without clinically evident cyanosis. From the hemodynamic point of view, TOF has two basic components viz., a large ventricular septal defect (VSD) and RVOT obstruction. Since the VSD is nonrestrictive, both right and left ventricle have the same systolic pressure. The configuration of the right ventricular pressure trace mimics that of the left ventricle [Figure 3]. The RVOT obstruction is often seen at more than one level, subvalvar, valvar and supravalvar. With the exception of absent pulmonary valve syndrome, infundibular stenosis is usually severe in TOF. The gradient across the RVOT varies with the change in right ventricular pressure which depends on the systemic pressure which in turn is determined by the systemic vascular resistance (SVR). Following a ventricular premature beat, the left ventricular pressure shows mild or no augmentation in the systolic pressure because of the reflex systemic vasodilatation. In cases of TOF with restrictive VSD, the RV systolic pressure shows post ectopic potentiation [Figure 4]. This phenomenon is typically absent in TOF as the right ventricle is influenced by the left ventricular pressure changes due the presence of unrestrictive VSD.

### RVOT pressure gradient vs resistance

RVOT obstruction is severe enough to keep the pulmonary artery pressure normal or below normal. Hence, a high RVOT pressure gradient is recorded. The systemic saturation is proportional to the pulmonary blood flow which in turn depends upon the relative resistance offered by the stenotic RVOT and the systemic circulation. A child with severe infundibular obstruction with good sized pulmonary annulus and branch pulmonary arteries has a relatively less resistance across the RVOT resulting in good pulmonary blood flow and remaining pink. However, the RVOT stenosis, as measured by Doppler gradient or at cardiac catheterization would be severe. On the other hand, a child with severe infundibular obstruction with severe valvar pulmonary stenosis, narrow annulus and/or small branch pulmonary arteries has a much higher RVOT resistance despite having a similar pressure gradient. In such a child, the pulmonary blood flow will be significantly reduced and most of the right ventricular blood would flow into aorta resulting in severe cyanosis.

### Effect of restrictive ventricular septal defect

In TOF, right ventricular pressure does not exceed the systemic level because of the unrestrictive VSD. However, in a small percentage of children, flow across the VSD may be restricted by the accessory tricuspid valve tissue, tricuspid valve vegetation or a prolapsed aortic valve. These children have supra systemic right ventricular pressure and hence record a much higher Doppler gradient. This suprasystemic RV pressure helps to overcome the RVOT resistance better, resulting in higher pulmonary blood flow and systemic saturation. The right atrial pressure trace shows a prominent ‘a’ wave which is a hallmark of restrictive VSD. The right ventricular pressure trace shows a characteristic ‘isosceles triangle’ pattern against a rectangular shape seen those with non-restrictive VSD [Figure 3 and 4]. Unlike the RV with nonrestrictive VSD, the one with restrictive VSD is more prone to failure.

### Effect of systemic vascular resistance

Right ventricular pressure is directly influenced by SVR due to large VSD. A reduction in SVR as seen during a febrile episode or during induction of anesthesia reduces the right ventricular pressure and hence reduces the antegrade flow into the pulmonary artery. This mechanism can explain why a cyanotic spell occurs even in a child of TOF with mild basal systemic desaturation.[1] Following balloon pulmonary valvotomy in TOF, the recorded RVOT gradient can occasionally be higher than the basal gradient despite improvement in systemic saturation.[2] This does not imply a failure of the procedure but signifies an increase in the SVR resulting in increased right ventricular pressure.

### Assessment of RVOT obstruction

**Figure 5:**
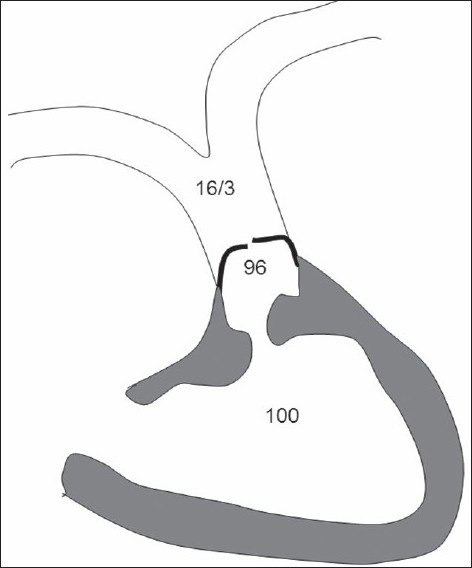
Pressures recorded at various levels in TOF with infundibular and valvar stenosis

Accurate assessment of the degree of stenosis at each level of obstruction is not possible by Doppler or cardiac catheterization. In the presence of distal obstruction, the obstruction at the proximal level is underestimated.[3] Figure 5 shows the pressures recorded in a case of TOF with subvalvar and valvar obstructions. The pressure in the subvalvar region is elevated so that blood is propelled across the more distal obstruction at the valvular level. Hence the pressure gradient at the infundibular level is small. This proximal gradient is unmasked when the distal obstruction is relieved by valvotomy. For the same reason, following balloon pulmonary valvotomy, there is no reduction in the overall RVOT pressure gradient in TOF. As seen in our case, there was a marginal reduction in the RVOT gradient after valvotomy. However, there was a significant reduction in the RVOT resistance resulting in increased pulmonary blood flow and prompt increase in the systemic saturation.

### TOF with mild pulmonary stenosis

A mild to moderate RVOT gradient is recorded in neonates with other features consistent with the diagnosis of TOF. This is due to two reasons. As the newborns have high pulmonary artery pressure, the recorded RVOT gradient is small despite a severe obstruction. This results in spurious conclusion that the obstruction is mild. The gradient increases over the first 2 to 3 weeks as a consequence of falling pulmonary vascular resistance. For the same reason, the measured gradient is low in patients with a large aorto pulmonary communication e.g. Pott's shunt or aortopulmonary window which increases pulmonary artery pressure.[4] In some neonates, the infundibular obstruction is actually mild in the newborn period. The progressive hypertrophy of the conal septum and the infundibulum increases the obstruction over a period of time resulting in a higher gradient recorded during the follow up. In children with absent pulmonary valve syndrome, the resistance to the RVOT flow is only at the level of the pulmonary annulus. The obstruction is variable from being mild to severe. With mild obstruction, there is increased pulmonary blood flow and the pulmonary artery pressure may be actually elevated to moderate degree despite a gradient being recorded across the RVOT (PS – PH situation).

### Ratio of pulmonary blood flow to systemic flow in TOF

In most children with TOF, the systemic blood flow (QS) is higher than normal as a compensatory mechanism for the low systemic saturation resulting in low arterio venous oxygen difference. This partly accounts for the enlarged aorta in these children. The pulmonary blood flow (QP) is usually lesser than QS, the difference accounting for the right to left shunt. In children with pink TOF, absent pulmonary valve syndrome and following aorto pulmonary shunts, the QP is higher than the QS. Hence, the pulmonary blood flow can be reduced, normal or higher than normal in TOF depending on various factors.

### Hemodynamics of heart failure in TOF

Heart failure is a rare occurrence in TOF. It is at times seen in children with increased pulmonary blood flow, viz., following a large aorto pulmonary shunt, presence of large patent ductus arteriosus or major aorto pulmonary collaterals. Less than 10% of adults with TOF develop systemic hypertension which increases the right ventricular pressure indirectly thus maintaining the antegrade flow across the RVOT. However, over a period of time this can result in biventricular dysfunction and failure. Similarly, anemia and aortic regurgitation increase the systolic pressure of both ventricles augmenting the pulmonary blood flow but adversely affecting their function in the long run.
